# A Binary Correlation-Based Ultrasonic Ranging System with Cross-Measurement Interference Mitigation

**DOI:** 10.3390/s26030834

**Published:** 2026-01-27

**Authors:** Ashish Mahanta, Gil Powers, Dulini H. S. Hewage, Jacob Powers, Haibo Wang

**Affiliations:** School of Electrical, Computer, and Biomedical Engineering, Southern Illinois University, Carbondale, IL 62901, USA; ashish.mahanta@siu.edu (A.M.); gil.powers@siu.edu (G.P.); dulinihimeka.sellahewage@siu.edu (D.H.S.H.); jacob.powers@siu.edu (J.P.)

**Keywords:** ultrasonic ranging, time of flight measurement, coherent detection, frequency modulation, phase modulation, FPGA implementation

## Abstract

This work presents a binary correlation-based ultrasonic ranging system that supports versatile signal modulation schemes. It transmits ultrasonic waves with distinct characteristics in consecutive measurement operations and employs a set of signal detectors to identify the originating excitation of the received echo signals, thereby effectively mitigating cross-measurement interference. Design equations supporting diverse modulation schemes are presented, and the factors affecting system performance are discussed. Experimental results obtained with the developed hardware system demonstrate its ability to perform accurate measurements over extended distances and under low signal-to-noise ratio conditions. Furthermore, the system effectively distinguishes echo signals generated by different excitation waves.

## 1. Introduction

Ultrasonic ranging remains an attractive technology in various applications [1,2,3,4,5,6,7], including robotics, industrial automation, navigation, etc. Compared with optical and radio-frequency alternatives such as LiDAR, depth cameras, and radar, ultrasound technology offers two primary advantages: environmental robustness and cost-effectiveness. Unlike optical sensors, which can be blinded by ambient light saturation or rendered ineffective by darkness, ultrasonic sensors are impervious to extreme lighting conditions. Furthermore, ultrasound is uniquely capable of detecting transparent surfaces, such as glass windows, and functions reliably in the presence of airborne particulates like dust and smoke, which frequently impair laser-based systems [7,8]. Additionally, the relatively low propagation speed of sound compared to electromagnetic waves allows for high-resolution Time of Flight (ToF) measurements using simple, low-cost hardware [9], making it an ideal solution for precise short-range applications where radar may be prohibitively complex or expensive.

However, deploying ultrasonic systems in complex environments that contain many acoustic reflecting objects at various distances presents significant challenges, primarily due to interferences between echoes originating from different transmitted signals and reflected by objects at different distances [10]. For example, an earlier-emitted excitation signal may return later than the echo of a subsequently transmitted signal if it is reflected from an object at a greater distance. This is often referred to as cross-measurement interference. Such interference can confuse the ranging system and lead to erroneous results [11,12]. This problem is further exacerbated by the demand for ultrasonic ranging systems with higher update rates.

To tackle this challenge, this work proposes to tag the acoustic waves emitted in consecutive measurements with different characteristics, such as different modulation or chirp patterns, or having slightly different frequencies. A group of signal detectors processes the received echo signals, with each detector tailored to a specific excitation signal. Thus, the originating excitation of each received signal can be unambiguously determined. Toward this goal, an FPGA-based design is proposed that accommodates diverse signal modulation schemes and facilitates efficient implementation of signal detectors. The proposed design is implemented on an FPGA development board and a custom PCB. Experimental results demonstrate that the system can maintain a measurement accuracy (standard deviation) of about 2 mm at distances up to 9.45 m, or in conditions where the signal to noise ratio (SNR) of the received signal is about 10 dB. It also shows the system can reliably distinguish echo signals generated by different excitations.

The rest of the paper is organized as follows: Section 2 briefly discusses related work. Section 3 explains the proposed method and its implementation, including the excitation signal generation circuit and signal detection circuit. System performance analysis is presented in Section 4, and experimental results are reported in Section 5. The paper concludes in Section 6.

## 2. Related Work

The Time of Flight method remains the most prevalent approach in ultrasonic ranging, wherein the accurate acquisition of timing information is critical for system performance. Among the various signal processing techniques available, the correlation method is regarded as the optimal estimation technique for ToF extraction [9,13]. Unlike simple Amplitude Threshold Methods (ATM), which are susceptible to noise peaks, the correlation method performs a cross-correlation calculation between the received echo and the transmitted signal, determining the flight time based on the maximum peak lag [14]. This approach offers superior accuracy; for instance, implementations utilizing frequency chirp signals (15–40 kHz) have demonstrated measurement accuracies of 1.2 mm within a 2300 mm range [15]. Furthermore, ToF estimation via signal envelope fitting has been reported to achieve sub-millimeter precision, reaching accuracies of 0.7 mm at distances up to 3000 mm [16]. However, a significant trade-off inherent to the correlation method is the increased computational load. Studies indicate that the processing time for correlation methods can be an order of magnitude higher than that of ATM; for example, processing times of 0.7 ms for correlation versus 0.07 ms for ATM have been observed using identical processing hardware [17].

To address the computational complexity of standard correlation and facilitate efficient real-time embedded implementation, binary techniques such as polarity correlation have been explored to reduce hardware overhead. In this approach, received signals are digitized into binary representations (logic 0 and 1) via infinite clipping to simplify the correlation function, effectively replacing complex multiplications with logical operations [18,19]. This method is particularly suitable for binary coded signals such as Binary Frequency Shift Keying (BFSK). Utilizing this concept, Nakahira et al. proposed a self-adapting sonar system with digital polarity correlators implemented on Field Programmable Gate Arrays (FPGAs). A key innovation in their work is the mitigation of the trade-off between near-field detection and far-field Signal-to-Noise Ratio (SNR); the system dynamically adjusts the transmission signal duration, specifically the number of wavelengths per bit, based on the detected range. While this variable-length pulse technique effectively suppresses cross-correlation peaks from multiple users and improves detection at longer distances, it relies on a discrete switching controller to modify the pulse pattern rather than employing a unified modulation scheme for simultaneous short- and long-range robustness [18].

Other hardware-optimized approaches include the work of Ureña et al., who developed an FPGA-based detector using 13-bit Barker codes. By optimizing the correlation process into a sequence of simple additions and subtractions, they achieved high precision with low-cost hardware [20]. Alternatively, Webster proposed a phase digitizing method to eliminate amplitude sensitivity errors. This technique estimates time delays of BFSK signals using zero-crossing timestamps rather than amplitude sampling [21].

Beyond computational efficiency, the robustness of ultrasonic ranging systems in reverberant environments relies heavily on the selection of modulation schemes to mitigate multipath interference and noise. Saad et al. demonstrated that utilizing wideband Frequency Hop Spread Spectrum (FHSS) signals combined with an earliest peak search algorithm significantly enhances robustness against multipath effects compared to narrowband methods [22]. In addition to timing accuracy, systems operating in crowded environments must mitigate crosstalk between sensors. Signal coding methods, such as Chirp, Gold codes, and M-sequences, are frequently employed to add distinguishing characteristics to transmitted waves, thereby improving SNR and minimizing crosstalk [23,24,25]. Specifically, M-sequences (pseudorandom sequences generated from linear feedback shift registers) combined with chirp signals have been shown to improve noise immunity and ToF estimation accuracy by ensuring the receiver can reliably identify the specific signature of the corresponding transmitter [26]. Regarding the choice of coding sequences, Benkhelifa et al. conducted a comparative study of m-sequences and Golay codes. They concluded that while Golay codes offer theoretical side-lobe cancellation, circular m-sequences provide superior SNR in practical, dynamic environments [27].

A novel zero-crossing-based ToF and center frequency estimation method for ultrasound signals was presented in [28]. It exploits the fact that zero-crossing times are concentrated around the maximum of the signal envelope. It offers simple processing and does not require reference signals; however, its performance is degraded when multiple signals overlap. Recently, artificial intelligence (AI) has emerged as a promising approach for improving ToF measurements, particularly in noisy and high-interference environments. In [10], an autoencoder-based deep learning neural network is used to analyze echo signals in the presence of obstacles around the object, whose distance is to be measured. Also, convolutional neural networks (CNNs) have been investigated to predict ultrasound ToF for non-destructive evaluation (NDE) [29] and ultrasonic computed tomography reconstructions [30]. Generally speaking, these techniques are computationally intensive and may limit their applicability in low-cost systems.

While existing research demonstrates the effectiveness of binary correlation and spread spectrum techniques in improving accuracy and SNR, there remains a need for a unified, hardware-efficient architecture capable of dynamically mitigating cross-measurement interference in high-speed sensing applications. Most prior works focus on optimizing static pulse patterns or rely on complex modulation schemes that strain low-power embedded resources. Building upon these foundations, this work advances the field by integrating flexible multi-mode modulation by combining frequency, phase, and chirping, with a streamlined binary detection architecture. Crucially, the proposed approach also accounts for the dynamic behavior of the transducer, affecting the accuracy and detection margins. This comprehensive design specifically targets the suppression of phantom echoes from preceding cycles while maintaining the computational simplicity required for real-time FPGA implementation.

## 3. Proposed Method and Implementation

**Figure 1:**
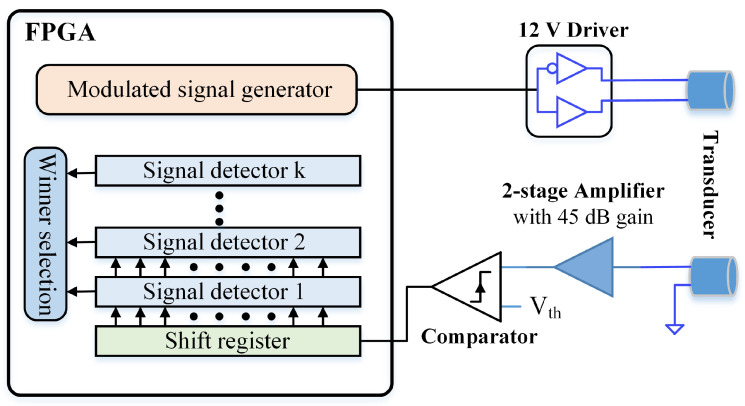
Proposed ultrasonic ranging system.

To mitigate cross-measurement interference, this work proposes to distinguish ultrasonic signals in consecutive measurements by assigning each one a unique characteristic parameter—such as frequency, modulation key, or chirping pattern. It also presents a hardware design that readily accommodates the requirements for generating and detecting these diverse signals. Figure 1 shows the block diagram of the proposed design. To avoid the complexity and noise susceptibility of conventional data-acquisition circuits (DAQ), the proposed design uses a high-speed comparator to digitize the phase of the received signal into binary bit sequences, which are subsequently processed through coherent detection techniques in the digital domain [9]. This dramatically simplifies the receiver-amplifier design by relaxing its gain and noise requirements and eliminating the need for advanced features such as programmable gain or additional filtering. To achieve high timing measurement precision, the comparator should operate at a high clock frequency. Alternatively, a time-to-digital converter (TDC) can be employed to improve timing measurement precision further [31].

Binary pulses from the modulated signal generation circuit drive the transmitting transducer. The circuit can generate signals with distinct characteristics in consecutive measurements, including varying frequencies, chirp patterns, and modulation schemes. Each signal detector is tailored to detect a specific type of transmitted signal. During measurement, all signal detectors simultaneously process the received signal, and the detector matched to the transmitted signal characteristics produces the highest correlation value. A winner-take-all circuit selects the detector with the maximum output, thereby identifying whether the echo originates from the current transmission or a previous measurement. The winner-take-all circuit can be implemented using a distributed architecture of digital comparators that perform simple bit-by-bit logic operations to efficiently locate the winner without the need for complex sorting algorithms [32,33]. The implementation of the modulated signal generation circuit and signal detector is discussed in the following subsections.

### 3.1. Excitation Signal Generation Circuit

**Figure 2:**
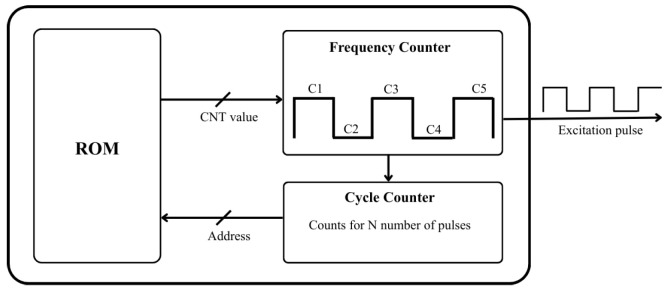
Excitation signal generation module.

As shown in Figure 2, the excitation signal generation circuit consists of a read-only memory (ROM), a frequency counter, and a cycle counter. The frequency counter initiates its countdown by loading an initial value from the ROM and toggles the excitation output when the count reaches zero. The cycle counter tracks how many cycles of the excitation signal has been generated and its output also serves as the ROM address input. By storing proper values in the ROM module, the circuit is capable of generating diverse excitation signals, including single-tone, chirp, frequency shifting key (FSK) and phase shifting key (PSK) modulated signals. The formulas for computing the ROM contents for different signal types are given below.

*Single-tone frequency signal:* In this case, the consecutive measurement operations use signals with different frequencies without any phase or frequency modulations. For a desired signal frequency *f*, the initial counter value *C* stored in the ROM is determined by:

(1) $$C=\left\lfloor \frac{f_{clk}}{2f}\right\rfloor$$

where, $f_{clk}$ is the counter clock frequency. The scaling factor 2 in the denominator is due to the fact that the initial counter value only corresponds to half of the signal cycle. The floor function rounds the value of the fraction expression down to the nearest integer. For a single-tone frequency signal with *N* cycles, the same C value computed by the above equation will be stored in 2N locations.

*FSK signal:* For each segment of a BFSK signal, its corresponding frequency initial counter value can be computed using Equation (1). These values are aggregated to generate the ROM content.

*PSK signal:* The signal maintains constant frequency *f* but experiences phase jumps at the transitions from one modulation key to another. For segments without a phase jump, their corresponding counter values are computed using Equation (1). For signal cycles where phase is modified, the associated counter values are given by:

(2) $$C_{k_{i}}^{1}=\left\lfloor \frac{f_{clk}}{2f}\left(1-\frac{\theta_{i}-\theta_{i-1}}{\pi }\right)\right\rfloor$$

where, $k_{i}$ represents the $i^{th}$ phase modulation key; $\theta_{i}$ and $\theta_{i-1}$ are the phase corresponding to keys $k_{i}$ and $k_{i-1}$. The superscript 1 of $C_{k_{i}}^{1}$ indicates that it is for the first signal cycle of the segments with a new modulation key. Changing the phase will either shorten or lengthen the first half-cycle of the signal that starts with a new phase. The terms inside the parentheses reflect this.

*Chirp signal:* Assume that the chirp signal frequency gradually varies from $f_{1}$ to $f_{2}$ over *N* signal cycles. Correspondingly, the counter initial values for the signal cycles during the chirping process should vary gradually from the value corresponding to $f_{1}$ to that corresponding to $f_{2}$. From Equation (1), the adjustment for the initial counter values from one signal cycle to the next can be derived as:

(3) $$\Delta C=\left(\frac{1}{N-1}\right)\frac{f_{clk}}{2}\left(\frac{1}{f_{2}}-\frac{1}{f_{1}}\right)$$

Thus, the ROM content for the $j^{th}$ cycle of the chirp signal is:

(4) $$C^{j}=\left\lfloor \frac{f_{clk}}{2f_{1}}+j\;\cdot \;\Delta C\right\rfloor$$

### 3.2. Signal Detection Circuit

**Figure 3:**
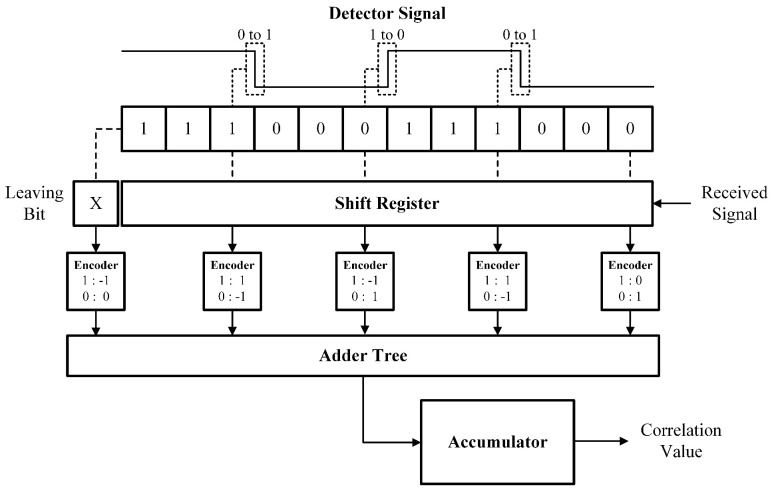
Block diagram of signal detection circuit.

The signal detector circuit computes the running correlation between its targeted pattern and the bit sequence in the shift register, as shown in Figure 3. Rather than computing the correlation bit by bit, the detector circuit implements a zero-crossing algorithm [20] that only monitors bit values at positions that the targeted signal (bit pattern to be detected) exhibits bit transitions. It takes advantage of the correlation value not being changed by bit shifting of the register in the regions where the targeted pattern has long runs of 1s or 0s. At the position that the targeted pattern transits from 0 to 1, the correlation needs to be incremented or decremented depending on the value of the received bit in that position: if the received bit is 1, the correlation changes by +1 because the bit now contributes; if the received bit is 0, the correlation changes by −1 because the bit no longer contributes. The same occurs for transitions from 1 to 0, with the correlation changing by +1 when the received bit is 0 and by −1 when the received bit is 1. The entering and leaving bits are also monitored to count their effect on correlation. To capture these changes, encoder circuits are placed at the start, transitional positions, and end, as shown in the figure. Each encoder will produce a partial value in two’s complement corresponding to the change that needs to occur in correlation, which is then summed with the others through an adder tree and accumulated every clock cycle to produce the current correlation output for the detector. Upon reset, the initial correlation is set to a precomputed value that is determined alongside the encoders by calculating the correlation whenever the shift register consists entirely of zeros.

Each detection circuit also includes a peak detector to record the peak correlation value and its corresponding time. Every clock cycle, the current correlation value and current counter value are fed into a comparison block. If the current correlation is greater than the currently stored peak value, the circuit will replace the stored peak value and its time with the results of this clock cycle. After a predetermined delay, the stored peak value will be fed to the winner-take-all circuit to compete for the winner. The winner will identify which transmitted signal corresponds to the received signal and its associated time will determine the time of flight of the ultrasound signal.

## 4. System Performance Analysis

### 4.1. Detection Margin for Signal Identification

To quantitatively assess the robustness with which the matching signal detector identifies the originating excitation of the received signal among competing detectors, we introduce *detection margin* (DM) as follows:

(5) $$DM=\frac{\rho^{+}-\rho^{-}}{\rho^{+}}$$

where $\rho^{+}$ and $\rho^{-}$ are peak correlations produced by the matching (positive) and an unmatching (negative) signal detectors, respectively. Higher DM values correspond to more robust signal identification.

The theoretical DM values for PSK signals can be derived as follows. Assume two signals, referred to as *a* and *b*, are modulated with their own sets of keys ${\left\{k_{i}^{a}\right\}}_{i=1}^{m}$ and ${\left\{k_{i}^{b}\right\}}_{i=1}^{m}$. The corresponding phases of their keys are denoted as $\theta_{i}^{a}$ and $\theta_{i}^{b}$. Since the two signals have the same frequency, the fraction of the region in which the two signals result in differing binary values under the $i^{th}$ modulation key is $\frac{\left|\theta_{i}^{a}-\theta_{i}^{b}\right|}{\pi }$. Averaging these results for all keys yields the DM value for the scenario in which detectors for signals *a* and *b* attempt to determine whether the received signal is an echo from either *a* or *b*. This is:

(6) $$DM=\frac{1}{\pi m}\sum_{i=1}^{m}\left|\theta_{i}^{a}-\theta_{i}^{b}\right|$$

This equation helps estimate detection margins when choosing phase modulation schemes. It indicates that large phase variations during modulation are preferred. However, the dynamic behavior of the transducers may constrain how quickly large phase variations can be achieved.

**Figure 4:**
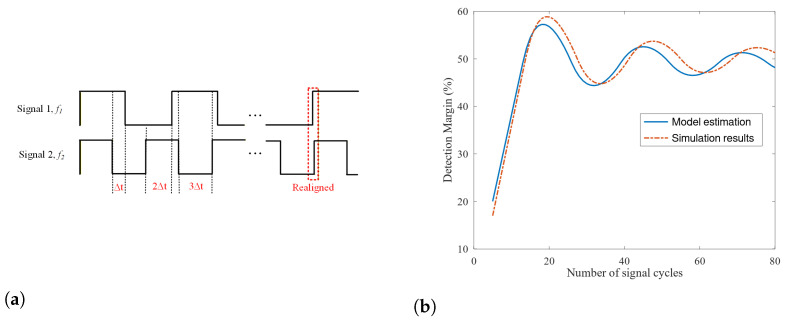
(**a**) Time of disagreement and realignment, (**b**) Simulated and modeled detection margin.

Estimating DM for single-tone signals is more complex. For two single-tone signals with frequencies $f_{1}$ and $f_{2}$, the durations in which the two signals result in different binary values are plotted in Figure 4a. For the convenience of discussion, such durations are referred to as time of disagreement (TD). The plot assumes $f_{1}<f_{2}$ and the two signals are aligned at the beginning. It indicates that the TD values increase by $\Delta =\frac{f_{2}-f_{1}}{2f_{2}f_{1}}$ every half-cycle of signal 1 before reaching its peak. After that, the TD values will decrease until the two signals are approximately aligned again. The number of signal cycles required to realign the two signals is around $P=\frac{f_{1}}{f_{2}-f_{1}}$.

Approximately, we assume the TD value reaches its peak around signal cycle $M=\left\lfloor P/2\right\rfloor$ and that the TD falling trajectory from signal cycle M+1 to *P* mirrors the TD rising trajectory from signal cycle 1 to *M*. Then, we have the following conclusions: (1) From cycles 1 to *M*, the TD values form an arithmetic series with an increasing common difference Δ every half cycle; (2) From cycles M+1 to *P*, the TD values form an arithmetic series with a decreasing common difference Δ every half cycle; (3) The accumulated TD values along its rising trajectory is about the same as that along its falling trajectory, which is S=Δ·M·(2M+1).

Assume the single-tone signal has *N* cycles. Let $\alpha =\left\lfloor \frac{N}{M}\right\rfloor$ and β=mod(N,M). The detection margin for detectors for such single-tone signals can be estimated by:

(7) $$DM=\left\{\begin{array}{cc} \frac{f_{1}}{N}\;\cdot \;\left(\alpha \;\cdot \;S+\Delta \;\cdot \;\beta \;\cdot \;(2\beta +1)\right), & \mathrm{if}\;\mathrm{mod}(\alpha ,2)=0,\\ \frac{f_{1}}{N}\;\cdot \;\left((\alpha +1)\;\cdot \;S-\Delta \;\cdot \;(M-\beta )\;\cdot \;(2(M-\beta )+1)\right), & \mathrm{otherwise}. \end{array}\right.$$

The case of mod(α,2)=0 corresponds to the scenarios in which the TD values per signal cycle increase during the final β cycles. The other case is for the scenarios that exhibit a decreasing trend for the TD values per signal cycle during the final β cycles. The scaling factor $f_{1}/N$ normalizes the accumulated TD values by the total duration of *N* signal cycles, thereby yielding the detection margin metric.

Figure 4b shows that the detection margins estimated by the above equation are close to those obtained from simulations, which emulate the operation of the proposed design. In the comparison, $f_{1}=39.7$ KHz and $f_{2}=41.2$ KHz. It also indicates that with adequate *N* values, the detection margin can reach about 50% despite the relatively small difference between $f_{1}$ and $f_{2}$. The detection margins for FSK or chirp signals are more complex to analyze, making it difficult to derive closed-form expressions. Simulations are the preferred approach to estimate detection margins for these two modulation methods.

### 4.2. Transducer Bandwidth Effects and Mitigation

**Figure 5:**
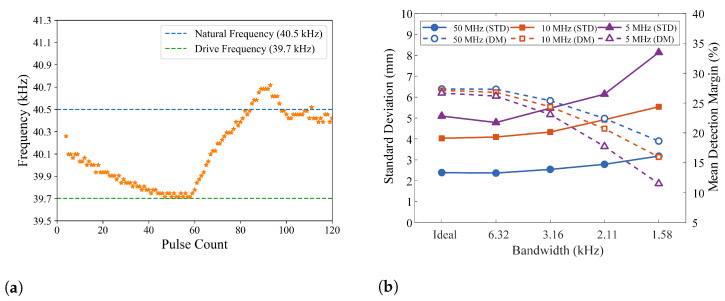
(**a**) Measured transient response of the transducer in response to acoustic signals, (**b**) Impact of transducer bandwidth on measurement accuracy and detection margin.

The 400S160 piezoelectric transducers used in this study have narrow bandwidth. As a result, the transient responses of ultrasound transducers can have notable impacts on both ranging accuracy and signal detection margins. Figure 5a plots the experimentally measured frequency trajectory of the transducer output at the onset of a 60-cycle acoustic wave. The transducer’s natural oscillation frequency is 40.5 KHz, and the frequency of the acoustic wave is 39.7 KHz. In this example, the acoustic signal frequency differs from the transducer’s natural resonance frequency to better represent scenarios where the system transmits multiple discrete frequencies, except in the PSK scheme. It takes approximately 40 cycles for the transducer output frequencies to settle at the driving signal frequency. After the acoustic wave ends, the transducer output frequencies return toward the transducer’s natural oscillation frequency.

The frequency transient response of the transducer is affected by the displacement characteristics of the transducer diaphragm, which can be modeled by a damped driven harmonic oscillator. This is:

(8) $$f\left(t\right)=f_{d}+\left(f_{n}-f_{d}\right)\;\cdot \;e^{-\frac{t}{\tau }}$$

where $f_{n}$ is the transducer’s natural oscillation frequency and $f_{d}$ denotes the frquency of the driving acoustic wave. The time constant τ is related to the transducer bandwidth BW as:

(9) $$\tau =\frac{1}{\pi \;\cdot \;BW}$$

The transducer used in the measurement has a bandwidth of 1.58 KHz, which leads to τ≈201μs and a settling time equivalent to 37 signal cycles for 1% settling accuracy. This is consistent with the measurement data plotted in Figure 5a.

Simulation results show that the transducer bandwidth can potentially affect both ranging accuracy and detection margins. Figure 5b plots the standard deviations of distance and the means of detection margins obtained from simulations with various transducer bandwidths. Note that the left end of the x-axis represents higher transducer bandwidths. The tick labeled “Ideal” corresponds to unlimited bandwidth, indicating that the transducer can instantaneously transition from its current frequency to the next desired frequency. The results are for scenarios of SNR = 0 dB with sampling rates of 5 MHz, 10 MHz, and 50 MHz. The bandwidth impact on measurement accuracy mainly manifests in scenarios with low SNR values, and it exacerbates with the decrease of sampling rate. The impact of transducer bandwidth on detection margins is more noticeable and less sensitive to SNR values.

As indicated above, using broadband transducers improves measurement accuracy and detection margins. However, in cases where air-coupled narrow-bandwidth transducers are preferred due to cost or acoustic output power considerations, increasing sampling rates helps mitigate the undesirable impact of limited bandwidth. Additionally, digital pre-distortion techniques can be employed during excitation signal generation to compensate for the transducer’s slow frequency transition. This involves gradually adjusting the excitation signal frequency to counteract the receiver’s slow frequency response modeled by the $\left(f_{n}-f_{d}\right)\;\cdot \;e^{-\frac{t}{\tau }}$ term in Equation (8). To achieve this, the counter values corresponding to individual signal cycles are computed as follows:

(10) $$C_{i}=\left\lfloor \frac{f_{clk}}{2\;\cdot \;(f_{d}-\left(f_{n}-f_{d}\right)\;\cdot \;e^{-\frac{t_{i}}{\tau }})}\right\rfloor$$

where, subscript *i* indexes the *i*th signal cycle, starting from either the signal onset or a frequency change in the FSK scheme, and $t_{i}=\frac{1}{f_{clk}}\;\cdot \;\sum_{k}^{i-1}C_{k}$. Since the excitation signal generation circuit stores counter values for individual signal cycles, this digital pre-distortion scheme can be easily accommodated in the proposed design.

## 5. Experimental Results

**Figure 6:**
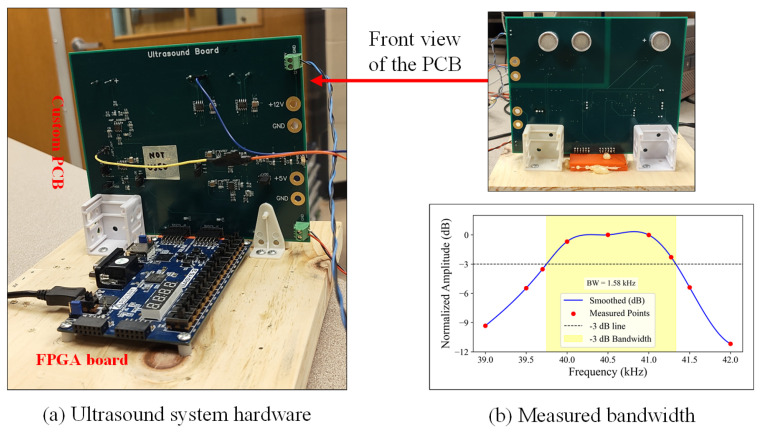
Developed ultrasonic ranging system.

The proposed design is implemented using an FPGA development board and a custom PCB, as shown in Figure 6. The ultrasound transducers used in the system are 400S160 piezoelectric sensors. The 3.3 V excitation signal from the FPGA is converted to 12 V level by an ADP3629 MOSFET driver. The receiver amplifier is a two-stage circuit with a gain of 45 dB, and its output is converted into binary sequences by an AD8611 comparator. The FGPA on the development board is an AMD Artix 7 XC7A35T device. The entire design operates with a 50 MHz clock.

Four modulation schemes, including FSK, PSK, chirp, and single-tone, were experimented with to evaluate the performance of the proposed design. The distinct characteristics of the signals associated with these modulation schemes are summarized in Table 1. Among the notations, *N* is the number of cycles of the signal; $F_{i}$ and $\Theta_{i}$ represent the sequences of frequency and phase values over the course of the signal cycles. The subscript *i* denotes different signals. The units of the frequency and phase are KHz and radian, respectively. Superscripts (N/2) or (N) indicate the value will repeat N/2 or *N* times. For chirp signals, the expression $f_{1}\overset{N/2}{\to }f_{2}$ represents that the signal frequency will gradually switch from $f_{1}$ to $f_{2}$ over N/2 cycles.

Measurement experiments were conducted in an auditorium and a plexiglass board with dimensions of 81 cm × 91 cm was placed in front of the ranging system at distances of 3m, 6m, and 9.45m (the longest distance allowed in the auditorium where experiments were conducted). At each distance, each modulation scheme was experimented with N=40,60,80. For each of these combinations, three measurement sessions, each consisting of 500 repeated measurements, were performed. The mean and standard deviation values for distance and detection margins reported in Table 2 were computed from these 1500 measurement results for each experiment setup. It should be noted that the reported distances are obtained by directly multiplying the speed of sound (343 m/s) with half the measured time of flight, without performing any calibration. Hence, the standard deviations of the measurement results serve as a more reliable metric for assessing the accuracy of the distance measurements. The achieved standard deviations outperform the existing designs that use comparators to digitize received signals. Also, the achieved measurement distance exceeds that of existing designs.

The detection margin, which is a distinctive feature of the proposed design, also demonstrates satisfactory performance. It shows single-tone signals yield the highest DM values. The difference between the DM values from experiments and those predicted by Equation (7) is likely due to signal noise and limited transducer bandwidth. PSK signals also achieve large detection margins, and the experimental results are pretty close to the DM value, 25%, computed using Equation (6). Chirp and FSK signals have relatively low detection margins, which is likely caused by the transducer’s slow transient response. With N=40, which is close to the transducer’s settling time of 37 cycles, the chirp signals exhibit a significantly reduced detection margin. As *N* increases to 60 and 80, allowing the transducer sufficient time to settle and track the frequency sweep, the detection margin improves substantially. Across all experiments, the originating pulses of the received echo signals were correctly identified, providing direct evidence of the robustness of the proposed method.

**Figure 7:**
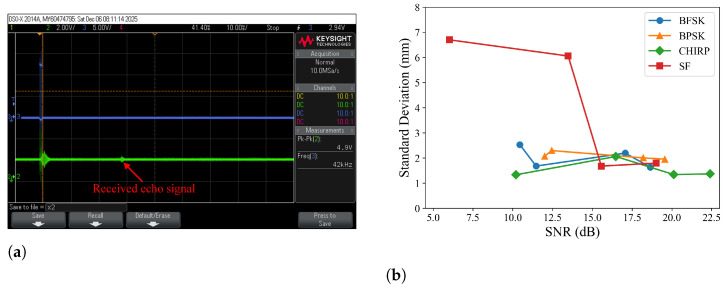
(**a**) Captured amplifier output with attenuated transmission power, (**b**) Standard deviation of measured distances vs. signal SNR values.

Experiments were also conducted to investigate the measurement accuracy with reduced strength of echo signals. We reduced the power supply voltage of the transducer driver to 9 V and covered the transmitting transducer with different sound-absorbing materials. The plexiglass board was placed 5 m away from the ranging system. Figure 7a shows a captured waveform from the amplifier output, which was used to compute signal SNR values. Figure 7b plots the standard deviation of measured distance versus signal SNR values. It shows that the system maintains high measurement accuracy even when the signal SNR approaches 10 dB. It also indicates that modulated signals (FSK, PSK, and chirp) outperform single-tone signals when the SNR values of received signals are low.

Further experiments were conducted using different objects or under different environmental conditions. Since the results in Figure 7b indicate that the single-tone scheme is the least robust among the four modulation schemes, these experiments focused on using the single-tone scheme. Table 3 presents the experimental results obtained with two different cardboard boxes positioned 4 m in front of the ranging system. The dimensions of the reflecting surfaces are listed in the second and third columns. The results in columns 4–7 indicate that the system achieves performance comparable to that observed in earlier experiments.

To demonstrate the system can operate in a noisy environment, experiments were conducted in a robotic lab with two Baxter robots turned on. These robots also emit ultrasound waves for detecting surrounding objects, which complicates the sensing environment. The plexiglass board was used in the experiment, and the obtained results are summarized in Table 4. These results demonstrate that the system maintains high ranging accuracy but experiences a reduced detection margin, decreasing from approximately 30% in earlier experiments to 16–18%. Nevertheless, the remaining detection margin is sufficient to distinguish pulses from different measurement cycles.

**Figure 8:**
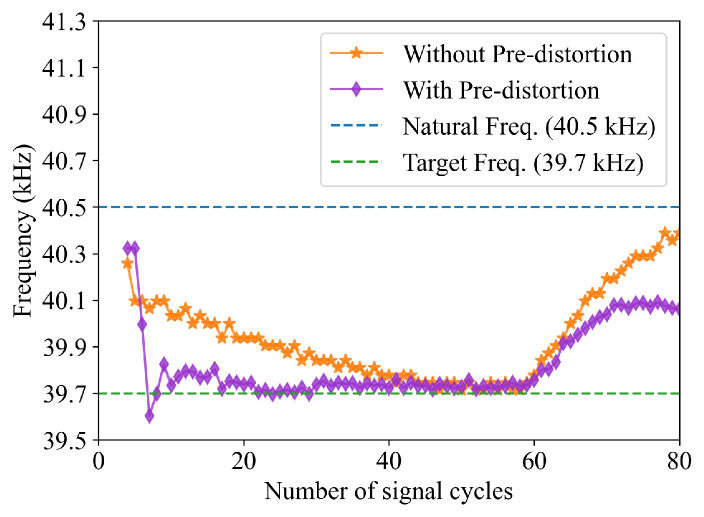
Frequency transient responses with and without excitation signal pre-distortion.

The digital pre-distortion scheme described by Equation (10) was implemented during excitation signal generation. This significantly improves the frequency tracking of the receiving transducer as shown in Figure 8. Without pre-distortion, the receiver signal’s frequency takes approximately 40 cycles to settle to the target frequency. With pre-distortion, the settling time is reduced to about 12 cycles. The improved frequency transient response allows the system to use fewer signal cycles in ranging operations. This is demonstrated by the experimental results in Table 5. The cardboard box 2 used in earlier experiments was positioned 4 m in front of the system, and the number of signal cycles is set to 20 in the ranging operations. Without pre-distortion, distance measurements fluctuate significantly and exhibit small detection margins. Applying digital pre-distortion significantly reduces the standard deviation of the measurement results and improves the detection margins, which demonstrates the effectiveness of the proposed pre-distortion scheme.

**Figure 9:**
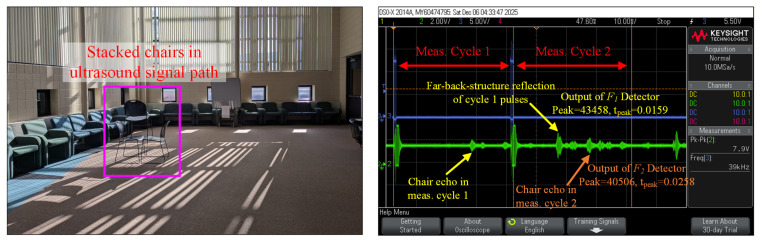
Demonstration of mitigating cross-measurement interference.

Finally, Figure 9 presents an experiment demonstrating how cross-measurement interference is mitigated with the proposed system. In the experiment, the system makes a measurement every 40 ms, corresponding to a 25 Hz update rate. A stacked chair structure was positioned approximately 4.5 m from the system, as shown in the photo portion of the figure. In the first measurement cycle, the system emits the FSK signal $F_{1}$ described in Table 1. The echo from the chair structure is received after approximately 25.8 ms, as indicated in the captured waveform. In this case, both the proposed system and conventional designs, which utilize thresholding or envelop detection methods to measure ToF [9], can correctly detect the chair structure and measure its distance. During the second measurement cycle, the system emits the FSK signal $F_{2}$ to distinguish it from the signal transmitted in the first measurement cycle. As shown in the captured waveform, two significant echoes are observed within the second measurement-cycle window, occurring at approximately 15.9 ms and 25.8 ms, respectively. A conventional thresholding or envelope detection based system would incorrectly interpret the echo at 15.9 ms as originating from an object located approximately 2.7 m in front of it. In reality, the signal at 15.9 ms is the echo of $F_{1}$ transmitted in the first measurement cycle and reflected by the far-end wall and furniture. In contrast, the proposed system employs $F_{1}$ and $F_{2}$ signal detectors to examine the received signal. The signal detector peak values and the times of reaching these peaks obtained from the FPGA circuit are annotated in the figure, illustrating how the system identifies that the echoes at 15.9 ms and 25.8 ms are originated by $F_{1}$ and $F_{2}$, respectively. As a result, the proposed system can effectively mitigate cross-measurement interference. Note that increasing the time intervals between consecutive measurements also helps reduce cross-measurement interference, but will decrease the update rate of the measurement system.

## 6. Conclusions

A low-cost, highly accurate, and versatile ultrasonic ranging system has been developed. By using binary correlation-based signal detection methods, the system simplifies the circuit design and relaxes the performance requirements for the receiver amplifier and data acquisition circuit. The proposed excitation generation and signal detection circuits support multiple signal modulation schemes, including FSK, PSK, chirp, and single-tone signals. During consecutive measurement operations, the system transmits ultrasonic waves with varying characteristics, and the signal detectors can accurately identify whether the received echoes arise from the current or prior excitation wave, enabling effective mitigation of cross-measurement interference. Experimental results indicate that the system can maintain a measurement accuracy (standard deviation) of about 2 mm at distances up to 9.45 m, or in conditions where the SNR of the received signal is about 10 dB.

## Figures and Tables

**Table 1:** Summary of signals used in experiments.

Mod. Method	Signal Design
FSK	$F_{1}=\left[39.7^{\left(N/2\right)},\;41.2^{\left(N/2\right)}\right]$, $F_{2}=\left[41.2^{\left(N/2\right)},\;39.7^{\left(N/2\right)}\right]$
PSK	$\Theta_{1}=\left[0^{\left(N/2\right)},\;{\left(\pi /4\right)}^{\left(N/2\right)}\right]$, $\Theta_{2}=\left[0^{\left(N/2\right)},\;{\left(-\pi /4\right)}^{\left(N/2\right)}\right]$
Chirp	$F_{1}=\left[39.7\overset{N/2}{\to }41.2\overset{N/2}{\to }39.7\right]$, $F_{2}=\left[41.2\overset{N/2}{\to }39.7\overset{N/2}{\to }41.2\right]$
Single-tone	$F_{1}=\left[41.2^{\left(N\right)}\right]$, $F_{2}=\left[39.7^{\left(N\right)}\right]$

**Table 2:** Measured distances and detection margins.

Modulation	Pulse Count	Distance Measurement and Detection Margin											
		Object Distance = 3 m				Object Distance = 6 m				Object Distance = 9.45 m			
		Distance		DM		Distance		DM		Distance		DM	
		$\mu_{\mathrm{dist}}$ (m)	$\sigma_{\mathrm{dist}}$ (mm)	$\mu_{\mathrm{DM}}$ (%)	$\sigma_{\mathrm{DM}}$ (%)	$\mu_{\mathrm{dist}}$ (m)	$\sigma_{\mathrm{dist}}$ (mm)	$\mu_{\mathrm{DM}}$ (%)	$\sigma_{\mathrm{DM}}$ (%)	$\mu_{\mathrm{dist}}$ (m)	$\sigma_{\mathrm{dist}}$ (mm)	$\mu_{\mathrm{DM}}$ (%)	$\sigma_{\mathrm{DM}}$ (%)
	40	3.01	0.23	4.44	0.04	6.00	0.16	7.67	0.37	9.39	1.23	8.42	0.36
FSK	60	3.03	2.09	10.40	0.21	6.01	1.49	14.12	0.28	9.40	0.42	12.74	0.31
	80	3.03	0.30	11.88	0.28	6.02	1.58	11.82	0.41	9.41	2.07	15.72	0.26
	40	3.00	2.21	18.92	0.55	6.00	2.19	17.40	1.17	9.40	3.09	16.82	0.64
PSK	60	3.01	0.89	19.36	0.18	5.99	2.51	18.32	1.26	9.39	4.37	17.86	1.68
	80	3.02	0.78	19.49	0.16	6.00	2.13	15.13	2.68	9.41	1.68	19.86	0.25
	40	3.02	1.04	1.24	0.19	6.02	0.66	5.30	0.60	9.42	1.69	6.67	1.04
Chirp	60	3.04	0.76	6.89	0.21	6.03	1.99	12.68	0.47	9.42	1.93	15.00	0.47
	80	3.04	0.31	14.86	0.53	6.03	1.52	14.32	0.60	9.42	2.20	16.26	0.38
	40	3.02	1.25	24.72	0.37	6.01	0.99	27.96	0.63	9.38	3.47	26.95	0.74
Single-tone	60	3.02	1.68	31.80	0.19	6.02	1.51	35.03	0.81	9.39	2.78	33.44	0.69
	80	3.02	0.46	35.18	0.14	6.02	1.02	37.36	0.35	9.42	2.46	35.42	0.47

**Table 3:** Experimental results with cardboard boxes of different sizes.

	Reflecting Surface Dimension		Distance		Detection Margin	
	Length (cm)	Width (cm)	$\mu_{\mathrm{dist}}$ (m)	$\sigma_{\mathrm{dist}}$ (mm)	$\mu_{\mathrm{DM}}$ (%)	$\sigma_{\mathrm{DM}}$ (%)
Cardboard Box 1	34	70	3.95	1.95	31.05	1.19
Cardboard Box 2	27	36	3.94	1.44	31.31	1.93

**Table 4:** Experimental results in a noisy environment.

Object Distance (m)	Measured Distance		Detection Margin	
	$\mu_{\mathrm{dist}}$ (m)	$\sigma_{\mathrm{dist}}$ (mm)	$\mu_{\mathrm{DM}}$ (%)	$\sigma_{\mathrm{DM}}$ (%)
3.5	3.51	0.99	16.14	4.06
4.5	4.51	2.26	18.33	5.36

**Table 5:** Measurement accuracy and detection margin with and without excitation signal pre-distortion.

	Distance Measurement		Detection Margin	
	$\mu_{\mathrm{dist}}$ (m)	$\sigma_{\mathrm{dist}}$ (mm)	$\mu_{\mathrm{DM}}$ (%)	$\sigma_{\mathrm{DM}}$ (%)
Without Pre-distortion	4.10	347.36	6.31	2.16
With Pre-distortion	3.91	3.15	11.30	1.59

## Data Availability

Data are contained within the article.
